# Antibodies against Ro52 in idiopathic inflammatory myopathies are associated with objective sicca symptoms

**DOI:** 10.3389/fimmu.2026.1841969

**Published:** 2026-06-09

**Authors:** Anna Meinecke, Benjamin Seeliger, Jonas C. Schupp, Thomas Skripuletz, Vega Gödecke, Marie-Therese Holzer, Diana Ernst, Torsten Witte

**Affiliations:** 1 Department of Rheumatology and Immunology, Hannover Medical School, Hannover, Germany; 2 Program of Hannover Medical School for Clinician Scientists (PRACTIS) Clinician Scientist Program, Dean’s Office for Academic Career Development, Hannover Medical School, Hannover, Germany; 3 Department of Respiratory Medicine and Infectious Diseases, Hannover Medical School, Hannover, Germany; 4 German Center for Lung Research (DZL), Biomedical Research in End-Stage and Obstructive Lung Disease Hannover (BREATH), Hannover, Germany; 5 Department of Clinical Airway Research, Fraunhofer Institute for Toxicology and Experimental Medicine (ITEM), Hannover, Germany; 6 Section of Pulmonary, Critical Care and Sleep Medicine, Yale School of Medicine, New Haven, CT, United States; 7 Department of Neurology, Hannover Medical School, Hannover, Germany; 8 Department of Nephrology, Hannover Medical School, Hannover, Germany; 9 Division of Rheumatology and Systemic Inflammatory Diseases, III Department of Medicine, University Medical Center Hamburg-Eppendorf, Hamburg, Germany

**Keywords:** idiopathic inflammatory myopathies (IIM), interdisciplinary research, interstitial lung disease (ILD), Ro52 antibody/TRIM21 antibody, Sjögren disease

## Abstract

Autoantibodies against Ro52 are not only detected in Sjögren’s disease (SjD), but also in idiopathic inflammatory myopathies, where they correlate with the incidence and severity of interstitial lung disease (ILD). It is still unclear whether antibodies against Ro52 are associated with further clinical manifestations in idiopathic inflammatory myopathies, as reduced tear and saliva production as a sign of associated SjD. Thus, this study aimed to determine the prevalence of objective SjD signs in Ro52-positive patients with idiopathic inflammatory myopathies, as well as the prevalence, characteristics, and clinical associations of ILD. A retrospective data analysis of patients from the Departments for Rheumatology, Nephrology, Neurology and Respiratory Medicine at Hannover Medical School, Germany was performed, in which a myositis immunoblot was determined between 2018 and 2024. Out of these patients, a total of 97 patients who were diagnosed with idiopathic inflammatory myopathy were included in the analysis. Overall, antibodies against Ro52 were detected in the myositis immunoblot in 46 of 97 patients (47%), whereas SSA antibodies were only detectable in 37 of 91 patients (41%) in the ELISA test. The detection of anti-Ro52 antibodies was associated with ILD (OR 3.5; p = 0.004). In addition, the presence of anti-Ro52 antibodies correlated with objective sicca symptoms, detected by reduced saliva and/or tear production in the Saxon or Schirmer test (OR 6.3; p=0.026). This study revealed in patients with idiopathic inflammatory myopathies, anti-Ro52 antibodies are associated both with the occurrence of interstitial lung disease and with reduced saliva and tear production. Thus, they mark associated SjD in idiopathic inflammatory myopathy patients.

## Introduction

Idiopathic inflammatory myopathies are a heterogeneous group of autoimmune disorders characterized by muscle inflammation, leading to muscle weakness, elevated creatine kinase levels, and extramuscular manifestations such as interstitial lung disease (ILD) and skin rashes (e.g., Gottron’s papules, heliotrope rash) (1). Idiopathic inflammatory myopathies can be divided into the subgroups of dermatomyositis (DM), inclusion body myositis (IBM), polymyositis (PM), immune-mediated necrotizing myopathy (IMNM) and juvenile dermatomyositis (JDM) (2). A distinction between these groups is usually made on the basis of the clinical phenotype. Recently, Leclair et al. showed that these five subgroups, as well as two more (antisynthetase syndrome (ASyS) and overlap myositis (OM)), could be differentiated by their antibody profile, and that the subgroups display different HLA profiles (3). Idiopathic inflammatory myopathies can present with a variety of symptoms. Typically, these are characterized by myositis, but other organ manifestations can also develop and may occur without overt myositis, such as ILD, skin involvement, or arthritis (4).

Myositis-specific and myositis-associated antibodies are present in around 75% of patients with idiopathic inflammatory myopathies, while only 20% of patients with positive myositis-specific antibodies or myositis-associated antibodies are diagnosed with idiopathic inflammatory myopathies (5, 6). The prognosis and life expectancy of idiopathic inflammatory myopathies varies depending on disease severity and organ involvement. Myositis-specific antibodies and myositis-associated antibodies can correlate with the clinical phenotype and therefore implicate conclusions on prognosis and management (7). While the group of myositis-specific antibodies includes autoantibodies that are unique to a diagnosis of idiopathic inflammatory myopathies, the group of myositis-associated antibodies does not contain any autoantibodies distinctive to the disease. Anti-Ro52, also known as TRIM21, is one of these most common myositis-associated antibodies. On a functional level, Ro52 acts as an E1 ligase, regulating type 1 interferon in addition to proinflammatory cytokines by ubiquitination (8). On a clinical level, anti-Ro52 is associated with various diseases, including Sjögrens`s disease (SjD) and myositis (e.g. Jo1-positive ASyS) (9). In idiopathic inflammatory myopathies, positivity of Ro52 correlates with the incidence and severity of ILD (10–12).

Additionally, Ro52, a 52-kDa protein, is one of the two most significant SSA/Ro isoforms, alongside Ro60, a 60-kDa protein. Although both SSA/Ro antibodies (Ro52/Ro60) are serological diagnostic for SjD, recent studies have shown that anti-Ro60 and anti-Ro52 positivity may be associated with different clinical phenotypes (13).

Despite the existing knowledge, the full clinical implications of Ro52 in patients with idiopathic inflammatory myopathy, except for the higher prevalence of ILD, remain to be elucidated. The association between Ro52 antibodies and the presence of additional symptoms, such as reduced tear and saliva production, remains uncertain. These symptoms could be indicative of concurrent SjD.

Thus, the primary aim was to assess the prevalence of objective SjD signs such as sicca symptoms in anti-Ro52positive patients with idiopathic inflammatory myopathies. Secondary endpoints included the prevalence and characteristics of ILD and associations with clinical phenotypes.

## Methods

### Study design

We performed a retrospective analysis of patients’ data from the Departments of Rheumatology, Nephrology, Neurology and Respiratory Medicine and Infectious Diseases at Hannover Medical School in which patients aged ≥18 years with a positive MSA/MAA antibody (by line blot, Euroimmun, Lübeck), measured between 01/01/2018 and 31/12/2024, and the diagnosis of probable or definite idiopathic inflammatory myopathy were included (2). Hannover Medical School is a tertiary referral center located in Germany. The evaluation of symptoms and the identification of antibodies occurred concurrently with the diagnosis. We evaluated muscle weakness by looking at the patient’s medical records. Where available, MRC-Sum-Score was used; otherwise, muscle involvement was determined by physician diagnosis (e.g., proximal weakness, functional limitations) as recorded in medical records. Dysphagia was evaluated via patient-reported symptoms, and, where available, barium swallow studies. Skin involvement was documented based on clinical examination (e.g. Gottron’s papules, heliotrope rash).

Patients with missing clinical data on diagnostic criteria were excluded. Those with other connective tissue diseases (e.g., systemic lupus erythematosus, systemic sclerosis) were also excluded unless idiopathic inflammatory myopathy was the primary diagnosis (17).

### Participants

**Figure 1:**
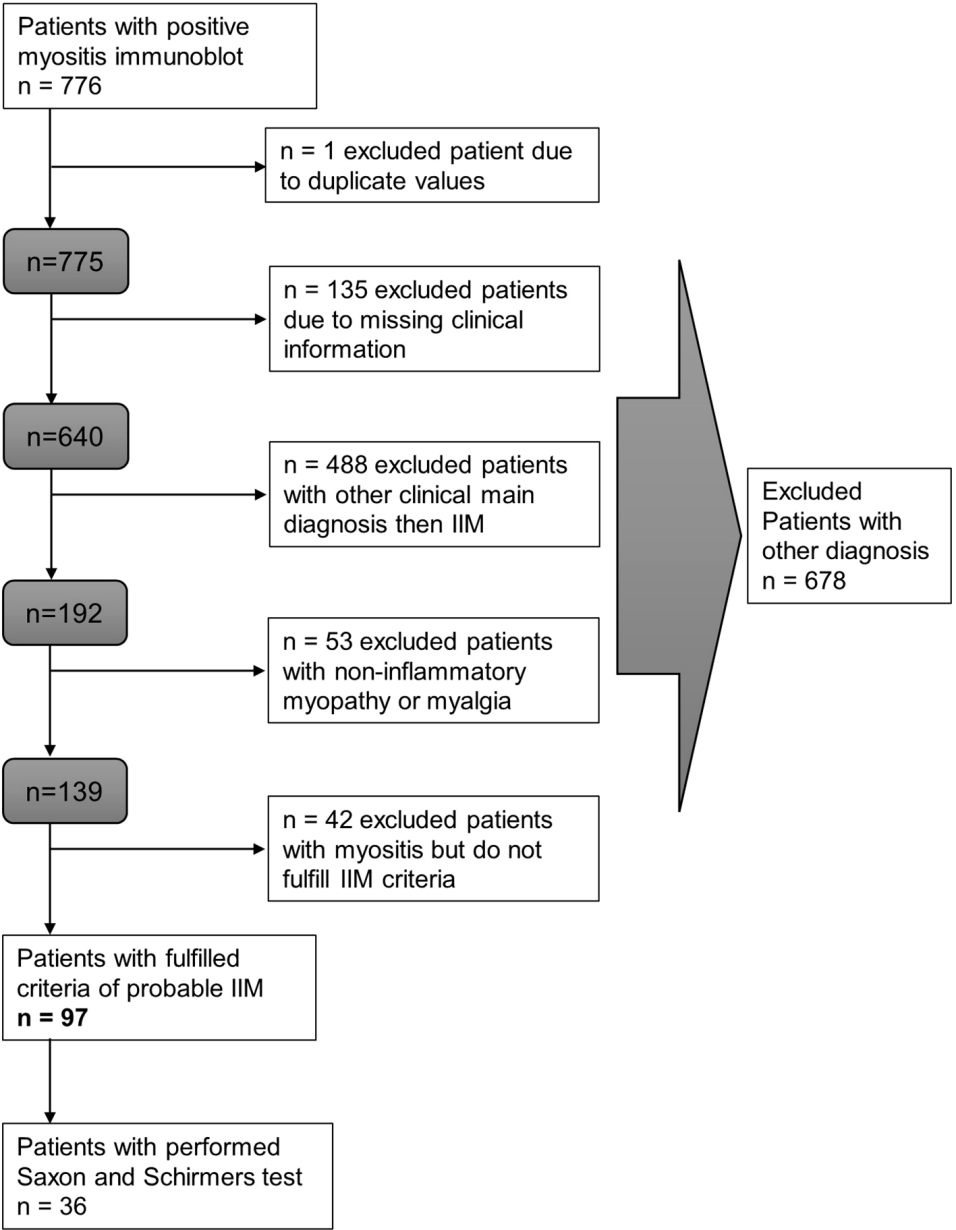
Study protocol. Initially, 776 patients were detected with positive Immunoblot. However, one patient was excluded due to duplicate data, 135 due to incomplete data, and 488 due to other clinical diagnoses. A further 53 were excluded due to a diagnosis of non-inflammatory myopathy or myalgia, and 42 due to symptoms that did not align with the IIM criteria. In conclusion, 97 patients were diagnosed with IIM, and 36 underwent a Saxon-Schirmer test.

In the examined timeframe, 776 patients exhibited positive results for myositis-specific and myositis-associated antibodies (**Figure 1**). In accordance with the established exclusion criteria, 679 patients were deemed ineligible for participation. Of these, 135 were excluded on the basis of incomplete clinical information. Of the 679 patients excluded from the study, 42 were diagnosed with myositis. However, these patients did not meet the ACR/EULAR criteria for probable or definite idiopathic inflammatory myopathy, and were therefore excluded from the study. Furthermore, 36 patients were excluded on the basis that they presented solely with myalgia, and 17 patients with a diagnosis of myopathy were excluded from the study. A total of 448 patients were excluded from the study on the basis that their primary diagnosis was not idiopathic inflammatory myopathy (**Figure 1**). When comparing the included and the excluded patients regarding age, sex and objective sicca symptoms, no significant differences were found. While anti-Ro52 is more present in the group of included patients (**Supplementary Figure 2****).**

Of the 97 patients included in our study, 49% (48 patients) met the criteria for probable idiopathic inflammatory myopathy, and 51% (49 patients) met the criteria for definite idiopathic inflammatory myopathy (**Table 1**). The mean age was 59.6 years with 57% of patients being female (see **Table 1**). Of the 97 patients, 52 (54%) were diagnosed with ILD (**Supplementary Table 3**).

**Table 1:** Patient characteristics of the overall population with fulfilled IIM criteria (n=97).

Clinical characteristics	Overall study population (n=97)	Anti-Ro52 positive (n=46)	Anti-Ro52 negative (n=51)	Fishers exact test Ro52 pos/neg, OR (95% CI)
Age Mean (± SD)	59.6 (± 13.3) years	57.4 (± 14.1) years	61.7 (± 12.3) years	p=0.115^#^
Sex Women	55/97 (56.7%)	23/46 (50.0%)	32/51 (62.7%)	p=0.225
ILD	52/97 (53.6%)	32/46 (69.6%)	20/51 (39.2%)	p=0.004, OR 3.5 [1.5;8.2]
Sjögren´s disease according to ACR/EULAR 2016 criteria	17/36 (47.2%)	15/22 (32.6%)	2/14 (14.3%)	p=0.002, OR 12.9 [2.2;73.6]
EULAR/ACR classification criteria IIM 2017				
Age at symptom onset ≥ 18 - < 40 years	8/97 (8.2%)	4/46 (8.7%)	4/51 (7.8%)	p=1.0
Age at symptom onset ≥ 40 years	88/97 (90.7%)	41/46 (89.1%)	47/51 (92.2%)	p=0.732
Objective symmetric muscle weakness UE	45/97 (46.4%)	19/46 (41.3%)	26/51 (51.0%)	p=0.416
Objective symmetric muscle weakness LE	49/97 (50.5%)	21/46 (45.7%)	28/51 (54.9%)	p=0.419
Weakness of neck flexors	3/97 (3.1%)	1/46 (2.2%)	2/51 (3.9%)	p=1.0
Predominantly proximal muscle weakness	42/97 (43.8%)	17/46 (37.0%)	26/51 (51.0%)	p=0.220
General skin manifestation	40/97 (41.2%)	20/46 (43.5%)	20/51 (39.2%)	p=0.685
Heliotropic rash	20/97 (20.6%)	8/26 (17.4%)	12/51 (23.5%)	p=0.616
Gottron’s papules	13/97 (13.4%)	6/46 (13.0%)	7/51 (13.7%)	p=1.0
Gottron’s sign	28/97 (28.9%)	16/46 (34.8%)	12/51 (23.5%)	p=0.265
Dysphagia or esophageal dysmotility	18/97 (18.6%)	11/46 (23.9%)	7/51 (13.7%)	p=0.296
Jo-1 autoantibody positivity*	44/97 (45.4%)	24/46 (52.2%)	20/51 (39.2%)	p=0.225
elevated CK or LDH or AST or ALT	90/97 (92.8%)	42/26 (91.3%)	48/51 (94.1%)	p=0.705
Muscle biopsy performed and conclusive with IIM	28/97 (28.9%)	8/46 (17.4%)	20/51 (39.2%)	p=0.025 OR 0.33 [0.1;0.8]
Probable IIM (Score ≥ 5.5 without biopsy, ≥ 6.7 with biopsy)	97/97 (100%)	46/46 (100%)	51/51 (100%)	–
Definitive IIM (Score ≥ 7.5 without biopsy, ≥ 8.7 with biopsy)	49/97 (50.5%)	28/46 (60.9%)	21/51 (41.2%)	p=0.068
Laboratory parameters				
Ro52 autoantibody positive (Myositisblot)	46/97 (47.4%)	46/46 (100%)	0/51 (0%)	–
Ro60 autoantibody positive (ELIA-Test)	7/74 (9.5%)	4/25 (16.0%)	3/49 (6.1%)	p=0.217
SSA autoantibody positive (ELIA-Test)	37/91 (40.7%)	34/43 (79.1%)	3/48 (6.3%)	p<0.001, OR 56.7 [14.2;225.3]
anti-alpha Fodrin IgA positive	11/72 (15.3%)	3/37 (8.1%)	8/35 (22.9%)	p=0.107
Elevated CK	60/97 (61.9%)	31/46 (67.4%)	29/51 (56.9%)	p=0.304
Elevated total IgG	19/79 (24.1%)	13/37 (35.1%)	6/42 (14.3%)	p=0.037, OR 3.3 [1.1;9.7]
Rheumatoid factor	17/83 (20.5%)	9/39 (23.1%)	8/44 (18.2%)	p=0.599

IIM, idiopathic inflammatory myositis; ILD, interstitial lung disease; LE, lower extremities; UE, upper extremities; SD, standard deviation; AB, antibodies; CK, creatine kinase; LDH, Lactate dehydrogenase; AST, aspartate aminotransferase; ALT, alanine aminotransferase. *either myositis immunoblot or EliA test; OR, Odds Ratio; CI, Confidence Interval; # unpaired t-test was performed for the normal-distributed variable Age.

### Assessments

#### Sicca measurements

The presence of objectively reduced saliva and tear production was determined with Saxon and Schirmer tests. Schirmer test was stated as pathological when less than 5mm/5 minutes and Saxon test less than 3.5 g in two minutes (14). The Saxon test was utilized to assess the unstimulated salivary flow. Due to the retrospective study design, Saxon and Schirmers tests were performed during the diagnosis of patients, with no follow-up measurements. SjD was diagnosed according to the ACR/EULAR 2016 criteria (15).

#### ILD definition

ILD was diagnosed based on the German Consensus guideline on the interdisciplinary diagnosis of interstitial lung diseases. According to this guideline, findings from high-resolution chest computed tomography (HRCT), pulmonary function testing and lung biopsy, as indicated following a multidisciplinary ILD board discussion (16). The reference values for pulmonary function tests are defined as follows: a vital capacity of less than 80% and a DLCO value of less than 80% of the normal value. This study did not evaluate the severity of ILD.

#### Laboratory tests

All the laboratory tests performed were part of the clinical routine. For the Myositis-specific antibodies and myositis-associated antibodies detection, a myositis line blot from Euroimmun (EUROLINE Myositis antigen test (Art.-No.: DL 1530-X G)), was performed. Ro52 was detected by the aforementioned myositis line blot while the measurement of antibodies against SSA (with the antigens Ro52 and Ro60) was carried out using an ELIA test (Thermofisher, Phadia™ 250 instrument, EliA Ro Well, Art.-No.: 14-5503-01). The supplementary files include a detailed description of the laboratory tests and antibodies used in the myositis line blot (see **Supplementary Table 1**).

### Statistical analysis

Statistical tests and descriptive analyses were performed using IBM SPSS Statistics version 29.0.1.0.

Participants were divided into two groups based on anti-Ro52 positivity. A Fisher’s exact test was then performed on these two groups to assess the effect of anti-Ro52 positivity on clinical parameters. The retrospective study was defined by the establishment of two endpoints: The present study investigates the association between anti-Ro52 and objective sicca symptoms, defined as pathological Saxon and/or Schirmer’s tests, as well as the association of anti-Ro52 and interstitial lung disease (ILD). It is imperative to note that the remaining correlations analyzed in this study are of an exploratory nature. Consequently, no Bonferroni correlation was performed. In addition to p-values, effect sizes were calculated to demonstrate the magnitude of the observed differences. The alluvial plot was created with R version 4.5.2 using the package ggalluvial and ggplot2.

### Ethical considerations

Following the declaration of Helsinki, approval of the local ethical committee was obtained (approval number 11147_BO_K_2023). Written informed consent for participation was not required from the participants or their legal guardians/next of kin, since this retrospective study only used pseudonymized data that does not allow individuals to be identified.

## Results

The results of the study demonstrated that 46% (45/97) of the patients exhibited symmetric muscle weakness of the upper extremity, while 51% (49/97) demonstrated symmetric muscle weakness of the lower extremity. Skin manifestations were present in 41% of the cases (40/97 patients) (**Table 1**). At the time of diagnosis, an elevated creatinine kinase (CK) was detectable in 60 out of 97 patients (62%), while 93% of the patients had either an elevated CK, lactate dehydrogenase (LDH), aspartate aminotransferase (AST) or alanine aminotransferase (ALT) (**Table 1**).

Overall, antibodies against Ro52 were detected in 46 out of 97 patients (47%) in the myositis blot, whereas Ro60 antibodies were only detected in 7 out of 74 (10%) in the ELISA test, and SSA in 37 out of 91 patients (41%). All patients with anti-Ro60 positivity were also anti-Ro52 positive. Anti-Ro52 positivity was associated with ILD (OR 3.5; p = 0.004) (**Table 2**). However, we detected no association between anti-Ro60 or SSA and ILD. In comparing the anti-Ro52 positive cohort with the anti-Ro52 negative cohort, it was additionally identified that there were significantly more cases of elevated IgG levels in patients who were positive for anti-Ro52 antibodies (p=0.037, OR 3.3), whilst a greater number of muscle biopsies were performed in those patients who were negative for anti-Ro52 antibodies (p=0.025, OR 0.33) (**Table 1****).**

**Table 2:** Patients with autoantibodies against Ro52 show objective sicca symptoms.

All patients with IIM:	Anti-Ro52 positive (n=46)	Anti-Ro52 negative (n=51)	Fishers exact test	OR (95% CI)
ILD	32/46 (69.6%)	20/51 (39.2%)	p=0.004	3.5 [1.5;8.2]
Performed Saxon-Schirmer test	22/46 (47.8%)	14/51 (27.5%)	p=0.058	
Pathologic Saxon and/or Schirmer test	19/22 (86.4%)	7/14 (50.0%)	p=0.026	6.3 [1.3;31.6]
Pathologic Saxon test (≤ 3.5 g)	6/22 (27.3%)	3/14 (21.4%)	p=1.0	
Pathologic Schirmer test (≤ 5 mm)	16/22 (72.7%)	8/14 (57.1%)	p=0.471	
Combination of ILD and pathologic Saxon and/or Schirmer test	12/22 (54.5%)	3/14 (21.4%)	p=0.083	
Patients with ILD and IIM:	Ro52 positive (n=32)	Ro52 negative (n=20)	Fishers exact test	OR
Pathologic Saxon and/or Schirmer test	12/15 (80.0%)	3/5 (60.0%)	p=0.560	
Pathologic Saxon test (≤ 3.5 g)	5/15 (33.3%)	1/5 (20.0%)	p=1.0	
Pathologic Schirmer test (≤ 5 mm)	9/15 (60.0%)	3/5 (60.0%)	p=1.0	

IIM, idiopathic inflammatory myositis; ILD, interstitial lung disease; OR, Odds Ratio; CI, Confidence Interval.

Additionally, anti-Ro52 presence correlated with objective sicca symptoms, detected by reduced saliva and/or tear production in the Saxon or Schirmer test, in the entire study population (OR 6.3; p = 0.026), though not in the subgroup of patients with ILD (**Table 2**). Similarly, we found an association between the presence of SSA antibodies and objective sicca symptoms in the entire study population (OR 7.56; p=0.025). However, we found no association between anti-Ro60 and objective sicca symptoms. Finally, 17 of 36 patients (47%) meet the ACR/EULAR 2016 criteria for SjD.

Moreover, 12 of 22 Ro52 positive patients (55%) showed an overlap of Sicca Symptoms and ILD while in the group of Ro52 negative patients only 3 of 9 patients (33%) have an overlap of Sicca Symptoms and ILD. However, these tendencies were not significant.

Furthermore, there was no statistically significant correlation between anti-Ro52 positivity and other clinical features, such as polyneuropathy, malignancy, arthritis or fatigue.

Of the 97 patients who fulfilled the criteria for idiopathic inflammatory myopathies, a total of 137 positive results for myositis-specific and myositis-associated antibodies were detected in these 97 patients, including 30 cases of multiple positive myositis immunoblots. Regarding the final study cohort of 97 IIM patients, anti-Ro52 was identified as the most prevalent myositis-associated antibody (34%), while Jo-1 was identified as the most prevalent myositis-specific antibody (23%) (**Supplementary Figure 1**). In 30 of 97 patients more than one myositis-specific antibodies or myositis-associated antibodies was detectable. The most frequent combination of myositis-specific antibodies or myositis-associated antibodies was anti-Ro52 and anti-Jo-1 (13/97 patients, 13%) followed by the combination of anti-Ro52 and anti-PL-12 and the combination of anti-Jo-1 and anti-PL-7 (both 3/97 patients, 3%), all of which had a primary diagnosis of ASyS.

The alluvial plot indicates the most frequent myositis-specific antibodies or myositis-associated antibodies for every patient in combination with the clinical main diagnosis and the fulfillment of the ACR/EULAR criteria for at least probable idiopathic inflammatory myopathy, which were met in 97 patients. Of the 679 patients excluded from the study, 18 were diagnosed with CIDP, 64 with primary SjD, 64 with other neurological diseases, 112 with other rheumatic diseases, and 104 with no autoimmune disease (**Supplementary Figure 2**).

## Discussion

This study demonstrates that objective signs of SjD, including reduced saliva or tear production, are present in 86.4% of anti-Ro52 positive patients. Notably, we also observed a high prevalence of ILD (53.6%), which was correlated with anti-Ro52 positivity. The aforementioned findings indicate the presence of a distinct clinical phenotype, thereby challenging the conventional separation of idiopathic inflammatory myopathy and SjD.

The observed positive correlation between anti-Ro52 positivity and the presence of ILD among our cohort is consistent with the findings of Valle et al., who also reported a correlation of anti-Ro52 positivity and more severe ILD cases (17). Our data also aligns with the results of Weng et al., who reported an eight-fold increased risk for ILD in idiopathic inflammatory myopathy patients with anti-Ro52 positivity (10). Additionally, it was shown that idiopathic inflammatory myopathy patients who were positive for anti-Ro52 had a higher risk to develop a rapidly progressive ILD (18). In patients with positivity for Jo-1, combined positivity with anti-Ro52 is associated with severe ILD and more frequent joint involvement (9).As the presence of an acute or subacute ILD worsens the prognosis of idiopathic inflammatory myopathy patients (19), it is essential to identify these disease manifestations at an early stage of the disease. The employment of autoantibodies, coupled with a more profound comprehension of the associated phenotype, has facilitated the identification of ILD. Recent guidelines on ILD in connective tissue diseases address the importance of early and regular screening for ILD especially in idiopathic inflammatory myopathy-risk groups like MDA-5 and antisynthetase-positive patients as well as prompt and sufficient therapeutic approaches (20).In addition to the presence of ILD, clinical factors like older age have been identified as significant factor for increased mortality rates in idiopathic inflammatory myopathy patients (21). Other negative factors associated with reduced survival were the presence of neoplasm and cardiac or respiratory muscle involvement (22).

In our study we demonstrated a correlation between anti-Ro52 positivity in idiopathic inflammatory myopathy patients and objective sicca symptoms detected by reduced tear and/or saliva production. Thus, we studied whether we could define a group of patients with idiopathic inflammatory myopathy and secondary SjD. To date, secondary SjD is known in systemic lupus erythematosus (SLE) where objective sicca symptoms and SS-A positivity are associated with fatigue (23). Uhlig et al. reported a prevalence of 7% for secondary SjD in rheumatoid arthritis (23). Regarding the coexistence of idiopathic inflammatory myopathy and secondary SjD, only a few case reports have been published concerning both autoimmune conditions (24). Additionally, the coexistence of idiopathic inflammatory myopathy and SjD has been reported in some patients (25).

Our findings support the existence of a distinct anti-Ro52 associated phenotype within idiopathic inflammatory myopathies, characterized by objective sicca symptoms and potential ILD. On the other hand, it is conceivable that a subset of patients may exhibit a primary SjD with a myositis phenotype as extraglandular manifestation as myositis is one of the ESSDAI domains used for clinically rating SjD. However, further longitudinal studies are needed to determine whether this represents a true overlap with SjD or a unique subtype of within idiopathic inflammatory myopathies. Similar to recent advances in characterizing overlap-myositis subtypes such as scleromyositis, further research could thus help identify common or distinct pathomechanisms of a SjD phenotype in idiopathic inflammatory myopathies (24). Furthermore, depicting SjD in idiopathic inflammatory myopathy might be relevant as SjD patients exhibit increased risk for hematooncological malignancies and screening is recommended regularly (25).

Our study demonstrated an association between anti-Ro52 positivity and ILD as well as an association between anti-Ro52 positivity and objective sicca symptoms, whereas anti-Ro60 positivity was not associated with these conditions. This finding is consistent with the observations of Lee et al., who reported different phenotypes in anti-Ro52-positive SjD patients compared to anti-Ro60-positive SjD patients (26). This finding supports the hypothesis that the prevalence of anti-Ro52- and anti-Ro60-positive patients differs across various autoimmune diseases, and that the associated disease phenotypes vary, as has been demonstrated, for example, in SjD or systemic lupus erythematosus (26, 27). Consequently, the implementation of routine, distinct testing for Ro52 and Ro60 antibodies would facilitate enhanced disease stratification, risk assessment, and clinical management.

A further question that remains unanswered is whether patients suffering from idiopathic inflammatory myopathy and associated SjD respond differently to therapies than patients with only idiopathic inflammatory myopathy. For patients with SjD and overlapping idiopathic inflammatory myopathy it is known that many exhibit a phenotype of mitochondrial pathology like inclusion body myositis (28), a myositis subtype usually responding worse or not at all to immunosuppressive treatment. Yet, amongst patients with e.g. inclusion body myositis, patients with SjD seem to respond better to immunosuppressive treatment than patients without associated SjD (29).

## Limitations

The study’s findings, implications and recommendations must be considered within the context of certain limitations. Initially, data collection was based on a monocentric study conducted at a tertiary referral center with a specialized ILD clinic in Germany. It is acknowledged that the included patients may not be representative of those attending external outpatient clinics. For instance, this may be evidenced by the higher frequency of ILD (53.6%) compared to metanalysis-based cohorts (26%) potentially inflating the observed association between Ro52 and ILD (30). Future studies should validate these findings in unselected IIM populations of idiopathic inflammatory myopathy patients.

Secondly, since this was a retrospective study, only a small proportion of idiopathic inflammatory myopathy patients underwent the Saxon-Schirmer`s test, as it is not part of the standard clinical routine for these patients. Thus, this could lead to selection bias among patients who were sent for the test. In addition, a higher proportion of anti-Ro52 positivity was observed in the included patient group (47.4%) compared to the excluded patients (28.5%), which might be due to the high overall prevalence of anti-Ro52 in IIM patients compared to other autoimmune diseases. Moreover, in certain instances, there was an absence of data for the workup of classification of idiopathic inflammatory myopathy, which resulted in the exclusion of these patients from the analysis. A further limitation of the study is the relatively small sample size, particularly in certain subgroup analyses. For instance, the limited number of positive anti-Ro60 results may result in non-significant outcomes for the correlation with objective sicca symptoms.

Due to the retrospective study design, there was no prospective evaluation of ILD based on the same criteria. Instead, the diagnosis was based on the clinical criteria of the German consensus guidelines, including HRCT and PFT. For the same reason, antibody reporting was only binary and lacked for titers in some cases. Future prospective studies should use uniform criteria for ILD and sicca evaluation. Furthermore, a limitation lays in the current classification criteria of idiopathic inflammatory myopathy. Latest research shows that certain idiopathic inflammatory myopathy subgroups are not sufficiently addressed by the classification criteria (31, 32). Yet, the classification criteria are standard for almost every clinical trial at the moment.

Consequently, further multicenter studies are required to investigate the impact of anti-Ro52 antibodies on the clinical phenotype and the clinical outcome in patients with idiopathic inflammatory myopathy in greater detail.

## Conclusion

In patients with idiopathic inflammatory myopathy, anti-Ro52 antibodies are associated both with the occurrence of interstitial lung disease and with reduced saliva and tear production in the Saxon and Schirmer tests. Thus, antibodies against Ro52 in idiopathic inflammatory myopathies serve as markers for associated SjD and have direct implications for clinical management. Screening for ILD and secondary hemato-oncological malignancies should be implemented in routine care for these idiopathic inflammatory myopathy patients.

## Data Availability

The data analyzed in this study is subject to the following licenses/restrictions: The datasets used in this study are not publicly available due to confidentiality a nd ethical restrictions, but are available from the corresponding author upon reasonable request. Requests to access these datasets should be directed to meinecke.anna@mh-hannover.de.
