# Supplementary material for: Antibodies against Ro52 in idiopathic inflammatory myopathies are associated with objective sicca symptoms
Source: Front Immunol. 2026 Jun 9;17:1841969. doi: 10.3389/fimmu.2026.1841969 (PMC13286919; doi:10.3389/fimmu.2026.1841969)
Supplement: Supplementary file 1 [file Table1.docx]

**Supplementary**

**Antibodies against Ro52 in idiopathic inflammatory myopathies are associated with objective Sicca symptoms**

A. Meinecke^1,2^, B. Seeliger^3, 4^, J. C. Schupp^3, 4, 5, 6^, T. Skripuletz^5^, V. Gödecke^6^, M.-T. Holzer^7^, D. Ernst^1^, T. Witte^1^

**Supplementary table 1: Laboratory tests**

| Laboratory test | Test information |
| --- | --- |
| IgG | Tina-quant IgG Gen.2, Cobas, Roche, Art.-No.: 08057915190 |
| Rheumatoid factor | Rheumatoid Factors II, Cobas, Roche, Art.-No.: 08058628190 |
| SSA/Ro | Thermofisher, Phadia™ 250 instrument, EliA Ro Well, Art.-No.: 14-5503-01 |
| Ro60 | Thermofisher, Phadia™ 250 instrument, EliA Ro Well, Art.-No.: 14-5525-01 |
| ALT | Alanine Aminotransferase acc. To IFCC, Cobas, Roche, Art.-No.: 05850797190 |
| AST | Aspartate Aminotransferase acc. To IFCC, Cobas, Roche, Art.-No.: 05850819190 |
| CK | Creatinin Kinase, Cobas, Roche, Art.-No.: 07190794190 |
| LDH | LDHI2, Lactate Dehydrogenase acc. To IFCC, Cobas, Roche, Art.-No.: 08057958190 |
| Myositis line blot | EUROLINE Myositis antigen test, Euroimmun, Art.-No.: DL 1530-X G  Included Antibodies: Mi-2 alpha, Mi-2 beta, TIF1g, MDA5, NXP2, SAE1, Ku, PM-Scl100, PM-Scl75, Jo-1, SRP, PL-7, PL-12, EJ, OJ, Ro-52 |

**Supplementary table 2: Comparison of included patients to excluded patients**

|  | **Included patients with IIM (n=97)** | **Excluded patients (n=678)** | **Fishers exact text** | **Mann Whithney test** |
| --- | --- | --- | --- | --- |
| **Age, mean ± SD** | 59.6 years +- 13.3 | 56.12 +-16.18 |  | p=0.1268 |
| **Sex, Women** | 55/97 (56.7%) | 352/678 (52.0%) | p=0.516 |  |
| **Ro52 positivity** | 46/97 (47.4%) | 193/678 (28.5%) | p=0.0002, OR 2.3 [1.4;3.6] |  |
| **Pathological Saxon and or Schirmers test** | 21/29 (72%) | 36/48 (75%) | p=1 | |
| ***Shapiro Wilk test indicated non-normal distributed data.** | | | | |

**Supplementary table 3: Overview of HRCT findings (n=52 patients with ILD pattern in HRCT)**

| HRCT pattern | Prevalence (%) |
| --- | --- |
| UIP | 7/52 (13.5%) |
| OP | 11/52 (21.2%) |
| NSIP | 21/52 (40.4%) |
| CPFE | 2/52 (3.8%) |
| Unclassified* (e.g. recurrent ground-glass opacities, reticulations, increased interstitial markings) | 11/52 (21.2%) |

*due to retrospective study design


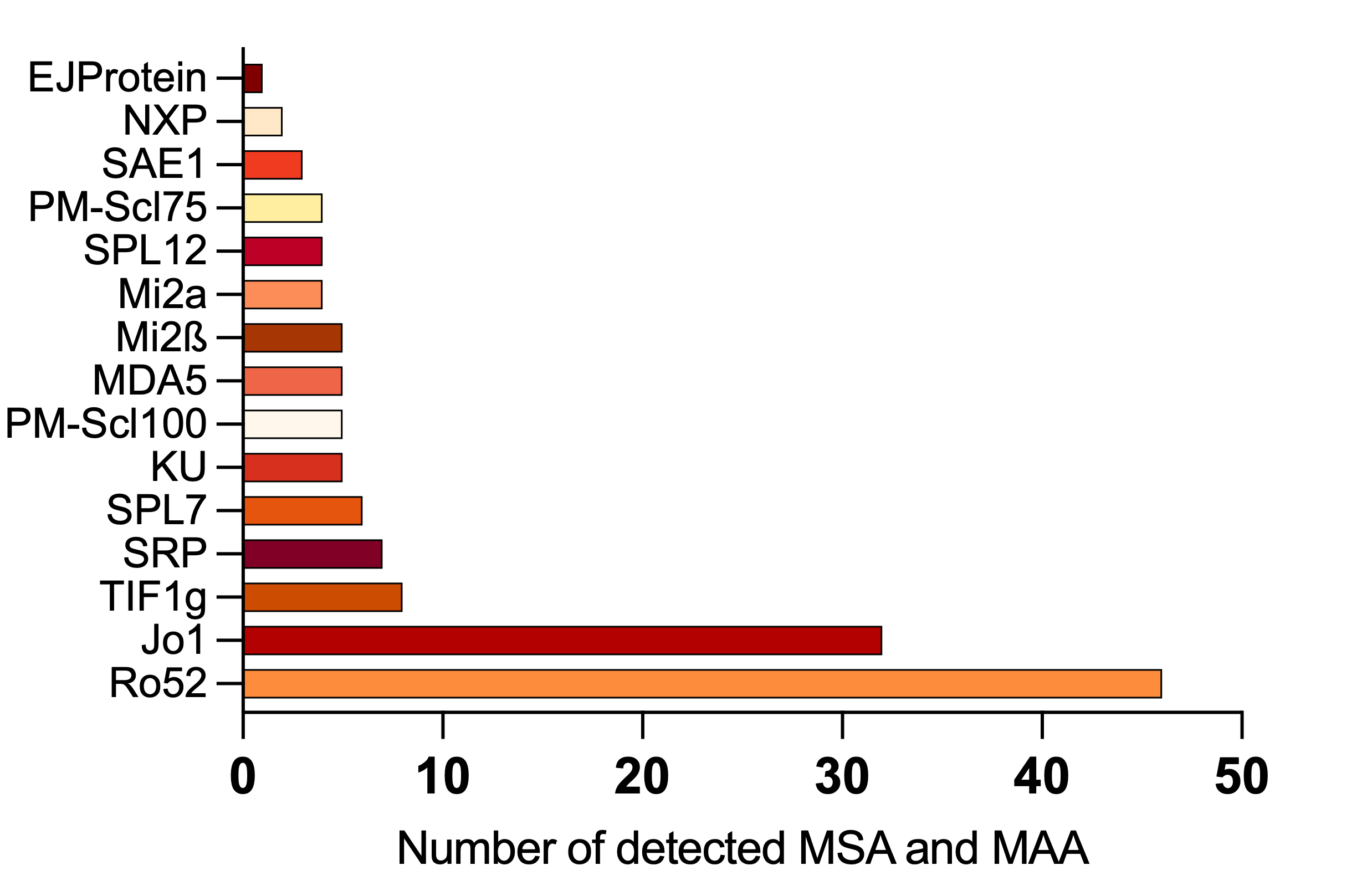


**Supplementary Figure 1: Prevalence of MSA and MAA**

Prevalence of myositis specific (MSA) and myositis associated antibodies (MAA) in our final study cohort, multiple antibody positivity possible (n=97 patients with 137 positive results for MSA or MAA).


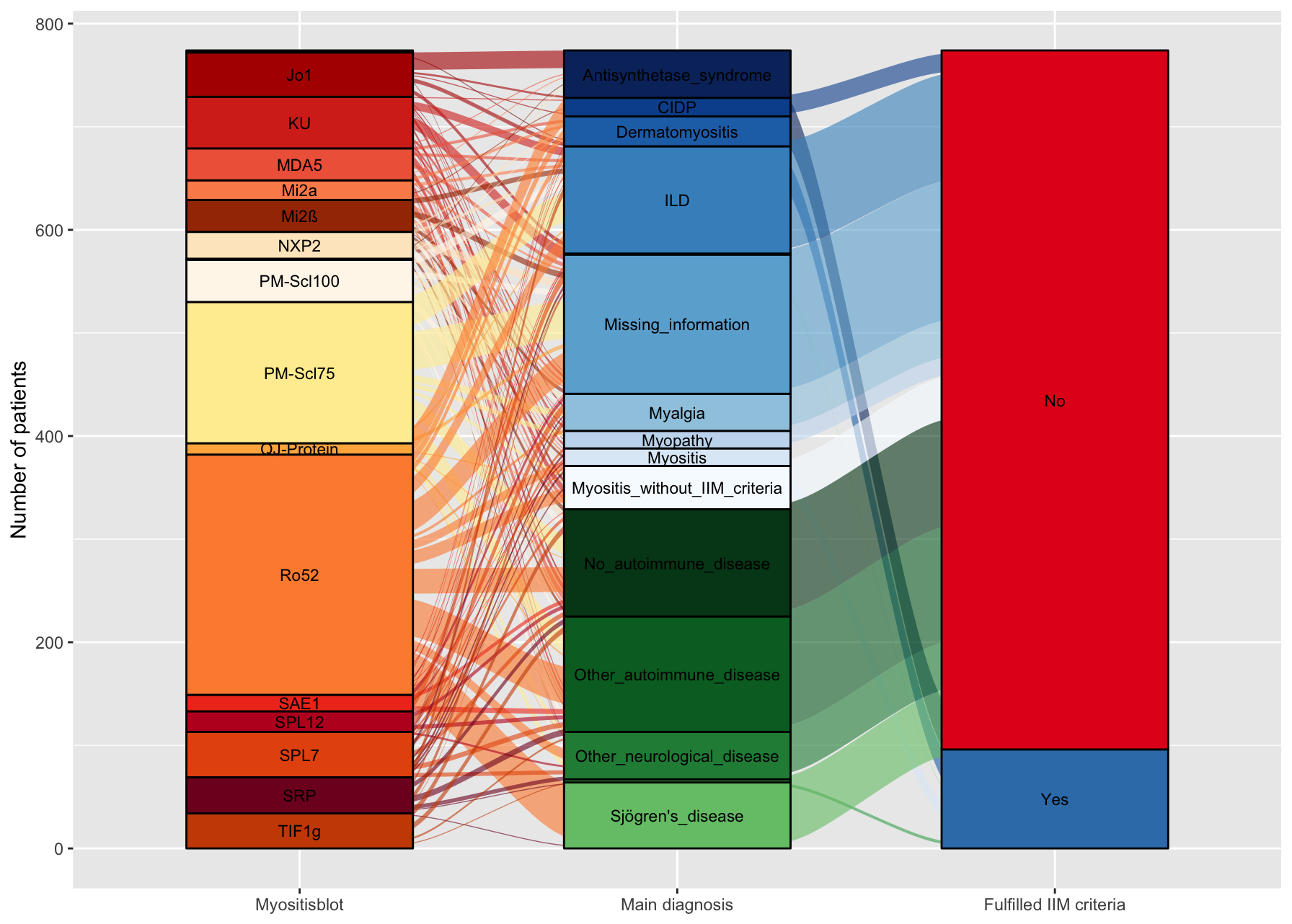


**Supplementary Figure 2: Alluvial Plot of antibody-positivity and the main diagnosis**

Alluvial Plot of the most abundant myositis antibody in each immunoblot, the main diagnosis and fulfillment of idiopathic inflammatory myopathy (IIM) criteria (n=776 patients).
